# May Renal Resistive Index Be an Early Predictive Tool of Postoperative Complications in Major Surgery? Preliminary Results

**DOI:** 10.1155/2014/917985

**Published:** 2014-05-20

**Authors:** Enrico Giustiniano, Massimo Meco, Emanuela Morenghi, Nadia Ruggieri, Daniele Cosseta, Silvia Cirri, Orazio Difrancesco, Paola Cosma Zito, Yari Gollo, Ferdinando Raimondi

**Affiliations:** ^1^Department of Anesthesia and Intensive Care Unit, Humanitas Research Hospital, Via Manzoni 56, 20089 Rozzano (Milano), Italy; ^2^Cardiac Surgery Anesthesia Unit, Sant'Ambrogio Clinical Institute, Milano, Italy; ^3^Biostatistic Unit, Humanitas Research Hospital, Rozzano (Milano), Italy

## Abstract

*Background.* Patients who undergo high-risk surgery represent a large amount of post-operative ICU-admissions. These patients are at high risk of experiencing postoperative complications. Renal Resistive Index was found to be related with renal dysfunction, hypertension, and posttraumatic hemorrhagic shock, probably due to vasoconstriction. We explored whether Renal Resistive Index (RRI), measured after awakening from general anesthesia, could have any relationship with postoperative complications. *Methods.* In our observational, stratified dual-center trial, we enrolled patients who underwent general anesthesia for high-risk major surgery. After awakening in recovery room (or during awakening period in subjects submitted to cardiac surgery) we measured RRI by echo-color-Doppler method. Primary endpoint was the association of altered RRI (>0.70) and outcome during the first postoperative week. *Results.* 205 patients were enrolled: 60 (29.3%) showed RRI > 0.70. The total rate of adverse event was 27 (18.6%) in RRI ≤ 0.7 group and 19 (31.7%) in RRI > 0.7 group (*P* = 0.042). Significant correlation between RRI > 0.70 and complications resulted in pneumonia (*P* = 0.016), septic shock (*P* = 0.003), and acute renal failure (*P* = 0.001) subgroups. Patients with RRI > 0.7 showed longer ICU stay (*P* = 0.001) and lasting of mechanical ventilation (*P* = 0.004). These results were confirmed in cardiothoracic surgery subgroup. RRI > 0.7 duplicates triplicates the risk of complications, both in general (OR 2.03 93 95% CI 1.02–4.02, *P* = 0.044) and in cardiothoracic (OR 2.62 95% CI 1.11–6.16, *P* = 0.027) population. Furthermore, we found RRI > 0.70 was associated with a triplicate risk of postoperative septic shock (OR 3.04, CI 95% 1.5–7.01; *P* = 0.002).

## 1. Introduction

Patients who undergo high-risk surgery represent a large amount of postoperative ICU-admissions. One of the most challenging issues is to identify the high-risk patient who should experience any complication in postoperative period and who could benefit from ICU surveillance and monitoring.

Many score systems developed aiming to quantify and predict the risk of a poor outcome, but none of these can be considered the best one. Further, several countries performed specific guidelines for ICU admission considering many aspects of the problem [1].

Renal resistive index (RRI = peak systolic velocity-end diastolic velocity/peak systolic velocity) consists of the measurement of renal arterial resistances to blood flow detected by echo-color-Doppler system and it is reliably correlated with kidney injuries and its severity [2–4]. In several trials, RRI showed a direct correlation with cardiovascular damage and seems to be an excellent indicator of prognosis in patients with severe hypertension [5, 6].

Renal resistive index could be applicable also in the monitoring of critical patients. Recently Bossard et al. studied patients at risk of renal insufficiency undergoing cardiac surgery. In these patients, authors calculated the RRI after patients entered intensive care unit (ICU). They found that renal resistive index is greater in patients who develop renal failure. In addition, the resistivity index correlates well with the severity of kidney damage, [7].

Darmon et al. studied renal resistivity index in patients admitted to ICU measuring in order to predict the onset of kidney failure during the course. Authors found that RRI was able to predict the onset of renal failure and the potential reversibility of the same [8]. Recently, Corradi et al. reported that RRI does not change in septic patients after fluid challenge, both in fluid-responder and no-responder subjects [9].

Furthermore, RRI may be an early independent factor detecting posttraumatic haemorrhagic shock in patients admitted to Emergency Department for traumatic injury [10]. Considering these findings and the fact that when important complications occur one of the main clinical signs is often the reduction of diuresis as a consequence of vasoconstrictive event in renal circulation, we explored whether RRI (detecting the renal circulation constrictive reaction) could be correlated with postoperative complications in major high-risk surgery, aiming to consider the parameter as a potential early predictor of outcome.

## 2. Material and Methods

The study was an observational phase II dual-center trial including patients who underwent major surgical operation, in accordance with the Helsinki Declaration. All subjects gave a written informed consent and the study was approved by the local Ethics Committee [*Avv. Pier Giuseppe Torrani President of Humanitas Research Hospital Ethics Committee, via Manzoni 56, 20089 Rozzano (Milano), Italy; Protocol Number of the authorization CE ICH-158/12; Date of approval: June 26th, 2012*].

The trial was performed according to STROBE statement for observational studies [11].

Primary endpoint was the association between altered RRI > 0.70 and complications occurring during the first postoperative week, but after 24 h from the end of surgery. We adopt 0.7 as cut-off value of RRI as it is reported in most of the published trials about detection of renal vasoconstriction by Doppler-ultrasound system.

Inclusion criteria were age > 18 years, any gender, elective cardiac and thoracic surgery, vascular, neurologic, and abdominal major surgery, intraoperative general anesthesia, and ability to give informed consent.

Exclusion criteria were total renal replace treatment (RRT) for bilateral nephrectomy or chronic kidney failure (serum Creatinine > 2 mg/dL), pregnancy, and emergency operation.

General anaesthesia started with Propofol 2.5 mg/kg + Fentanyl 1-2 mcg/kg. A gas mixture including air, oxygen (FiO_2_ 0.50), and sevoflurane 1-2% was administered for anaesthesia maintenance. No-depolarizing myorelaxant drug (Atracurium 0.5 mg/kg or cis-Atracurium 0.15 mg/kg boluses, and repeated top up doses as needing) was administered for tracheal intubation and as requested after patient was connected to mechanical ventilator (protective ventilation: tidal volume 6 mL/kg; respiratory rate 10–12 apm; peak respiratory pressure limit 35 cm H_2_O; positive end-expiratory pressure 5 cm H_2_O). Fentanyl 1-2 mcg/kg bolus was readministered after 30 and 60 minutes.

Intraoperative monitoring included invasive blood pressure (IBP), electrocardiogram (EKG) with ST-segment analysis, heart rate (HR), end-tidal carbon dioxide (EtCO_2_), central venous pressure (CVP), and central venous oxygen saturation (ScvO_2_) according to clinical judgment. Cardiac output (CO) and correlated parameters were monitored by FloTrac/VigleoTM (Edwards Lifescience, Irvine, CA) in thoracic surgery and by pulmonary artery catheter (PAC) in cardiac operations, depending on the habits of the two centers participating to the trial. These data were recorded hourly reporting the worst value observed. Furthermore, a blood gas analysis was sampled and fluid balance was computed.

Within 15 minutes, after the transfer of the patient to the recovery room (or during awakening period in subjects submitted to cardiac surgery) a skilled anaesthesiologist performed renal resistive index measurement by C5-2 convex probe of EnVisor C HD sonogram (PHILIPS Ultrasound, Bothell, WA, USA 98041). We measured RRI in triplicate and then the average value was recorded. A blood sample was collected to test serum creatinine, serum lactate, and B-type natriuretic peptide (BNP). Outcome data were recorded for the whole period of hospital staying reporting the incidence of any type of complications occurring during the first postoperative week. Furthermore, at the end of operation we calculated the Surgical APGAR Score (SAS) aiming to explore any correlation of this validated system and RRI about detection of postoperative morbidity [12].

Our analysis was performed not only over the whole sample population, but also in the subgroup of patients who underwent cardiothoracic surgery as their abdominal-splanchnic district was not surgically injured. In these cases our hypothesis was that the subgroup may provide results not altered by surgical manipulation that could provoke any renal vascular bed reaction.

## 3. Statistical Analysis 

This was an observational dual-center trial, investigating whether RRI > 0.7 was associated with worse postoperative outcome in high-risk major surgery, within 7 postoperative days.

Variables are expressed as number and percentage within a 95% confidence interval (CI), mean and standard deviation, or median and range, as appropriate. Differences between group RRI < 0.7 and RRI > 0.7 were explored with chi-square test, Fisher correction if necessary, or Wilcoxon test, as appropriate.

ROC analysis was performed as appropriate.

Relative to the study of association with the presence or absence of complications, all the independent variables which have been found to have a statistical association with a *P* < 0.1 were subjected to a multivariable logistic regression analysis.

A *P* < 0.05 was considered significant. Analyses were performed with Stata 11 Softwar—StataCorp. 4905 Lakeway Drive College Station, Texas 77845-4512 USA.

## 4. Results

From July 2012 to February 2013 we enrolled 218 consecutive patients submitted to major high-risk surgery. Out of the whole sample 13 patients were excluded due to incomplete data collected, resulting in a final sample of 205 patients. We recorded 49 cardiac surgery (23 valve substitution/plasty, 21 CABG (coronary artery bypass graft), and 5 CABG + valve substitution/plasty), 96 thoracic surgery (35 lung lobectomy, 23 oesophageal surgery, 15 atypical resection, 11 VATS (video assisted thoracoscopy), 5 pneumonectomy, 3 pleurectomy, 2 pneumopericardial phrenectomy, and 2 thymectomy), 42 abdominal surgery (15 liver resection, 10 gastroenteric/colon resection, 8 abdominal aortic aneurysmectomy, 7 pancreatic-duodenal resection, and 2 pancreatectomy/splenopancreatectomy) 16 brain neoplasm resection, and 2 carotid endarterectomy.

The whole sample populations were labeled as ASA 1–4.  Table 1 reports data about enrolled patients.

Among the preoperative risk factors, age, BMI, ASA grading, smoking, diabetes, serum creatinine, sonographic end-diastolic volume, and haematocrit resulted significantly correlated with postawakening RRI > 0.7. (Table 2). Surgical APGAR score (SAS) was not related with RRI score (*P* = 0.520). Patients who experienced any postoperative adverse event had a surgical APGAR score of 7 points, conversely 6 points in subjects who did not (*P* = 0.001); we did not observe any direct correlation between SAS and RRI.

None of the intraoperative differences between the two groups of RRI, regarding hemodynamics, blood-gas analysis (serum Lactate included), and fluid balance were found. Also pO_2_/FiO_2_ ratio difference, 314 (73–707) in RRI < 0.7 versus 294 (140–560) in RRI > 0.7, was not statistically significant (*P* = 0.088).

We did not observe any patient with signs of sepsis, severe sepsis, or septic shock at time of RRI measurement.

Out of the 205 patients, 60 (29.3%; CI 23.0%–35.5%) showed RRI > 0.70. The total rate of adverse event was 27 (18.6%; CI 12.2%–25.0%) in RRI < 0.7 group and 19 (31.7%; CI 19.5%–43.8%) in RRI > 0.7 group (*P* = 0.042).

Table 3 reports the outcome data in the two RRI groups. In the global population, significant correlation between RRI > 0.70 and complications resulted about pneumonia (*P* = 0.016), septic shock (*P* = 0.003), and acute renal failure (*P* = 0.001). ARDS and SIRS/sepsis did not reach statistical significance, although they seemed indicative of a higher frequency in the group with RRI > 0.7. Furthermore, patients with RRI > 0.7 resulted to have longer ICU stay (*P* = 0.001) and lasting of mechanical ventilation (*P* = 0.004).

Also in cardiothoracic population we observed an association between RRI and complications (*P* = 0.042). Significant correlation between RRI > 0.70 and complications resulted about pneumonia (*P* = 0.007), septic shock (*P* = 0.013), and acute renal failure (*P* = 0.008). Patients with RRI > 0.7 needed a longer ICU stay (*P* = 0.006) and mechanical ventilation (*P* = 0.001) and longer length of stay (*P* = 0.023).

Neither the general nor the cardiothoracic population showed difference regarding mortality.

**Figure 1:**
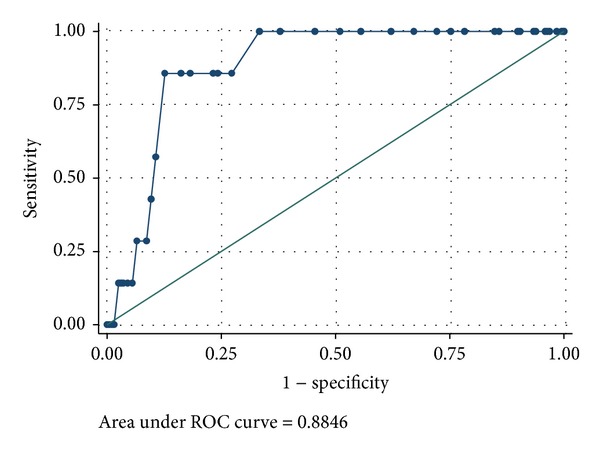
The ROC curve.

Furthermore, we explored the risk of complications both for septic shock and for pneumonia. Then we divided our population in five classes according to RRI ranges: (1) RRI < 0.6; (2) RRI 0.61–0.65; (3) RRI 0.66–0.70; (4) RRI 0.71–0.75; and (5) RRI > 0.75. Despite that we recorded an increasing risk of complications in corresponding increased-RRI range, differences were not statistically significant. But we noted that best predictive cut-off value might be RRI = 0.70 as OR showed the greatest incremental rising (Table 4), both in general and cardiothoracic population. The predictive effect of RRI ≥ 0.75 reached a good specificity and sensitivity (Figure 1). This result was confirmed for cardiothoracic subgroup too.

We did not report results about BNP because most of the enrolled patients did not receive the specific blood sampling as planned.

## 5. Discussion

Our study highlighted that patients who have postoperative RRI > 0.70 more frequently experienced complications compared to subjects with RRI < 0.70, with poor outcome in some cases.

The relationship between RRI > 0.70 and acute kidney injury has been demonstrated, and also our results were in agreement with previous literature reports [2–4]. But, even if this correlation could be understandable, as it relates to the injured organ, why RRI could predict an infectious disease like pneumonia with potential subsequent septic shock remains unclear to our mind. As septic shock is the final step of a continuous disease starting with infection and passing through a septic phase, RRI > 0.70 may be the epiphenomenon due to an early reactive renal vasoconstriction, [13].

Pinto et al., in an animal model, found that sepsis induces AKI by endothelial injury with hemodynamic dysfunction, release of inflammatory mediators, and reactive oxygen species (ROS) generation by tubular cells, in association with renal vasoconstriction due to hemodynamic and inflammatory disturbances, [14]. May RRI be an early detector of this systemic impairment in humans? If it would be so, RRI may result to be a helpful tool to detect systemic reaction in a very early phase. We also found a correlation between RRI > 0.70 and SIRS/Sepsis, but the result was near statistically significant. We consider that it may be due to a not-enough sample amplitude; we had a too low power (close to 18%) to see that difference as significant. Further, it was not a primary endpoint. In any case we recognize that it is a limit of our study that may be reported as a “preliminary results trial.”

Recently, Futier et al. showed that protective mechanical ventilation in a mixed surgical population reduces the risk of postoperative complications (pneumonia, SIRS, and septic shock) probably due to its lower stimulation of inflammatory cascade [15]. All our patients received protective mechanical ventilation in agreement with the ventilator setting adopted by Futier et al. Then we excluded such a confounding factor about the risk of postoperative adverse events.

Dewitte et al., found that RRI is unable to determine optimal mean arterial pressure at ICU admission of septic patients, [12]. In agreement with this result, we did not find any correlation of RRI with perioperative blood pressure even in septic patients. On the contrary, we observed some factors affecting RRI. Older patients more frequently showed higher RRI values. Also higher BMI was associated with RRI > 0.70, so as smoking and diabetes. We found correlation between lower end-diastolic volume and high RRI and worse RRI in subjects who had lower preoperative hematocrit. All these factors are included into the preanesthesia health assessment of the patients and it may explain the association of RRI > 0.70 and ASA 3-4 classification. Other factors (hypertension, previous cardiac disease, and albumin urinary loss) showed potential affecting action but it did not reach statistical significance.

Surgical APGAR Score (SAS) is a validated system to predict perioperative morbidity, based on three intraoperative variables: bleeding, the lowest MAP, and heart rate observed [16–18]. In our patients, SAS was lower in subjects who experienced a complicated outcome, but we did not find a significant parallelism of RRI with SAS. It may be due to MAP and heart rate; two factors that did not show any correlation with RRI measurement in our study.

In high-risk surgery, the identification of patients who may benefit from postoperative observation and monitoring is an important issue both for patient safety and for a correct management of ICU resources [1]. The identification of ICU-needing subjects is difficult to do preoperatively, but if we would find a helpful tool that may permit it in the early postoperative phase, it could be a good step forward. We are convinced that our study is far from the solving the problem, but we consider that it may be a starting point for further research.

Cardiothoracic subgroup of patients showed similar results. Then we may speculate that RRI is not affected by surgical manipulation. Nonetheless it could be another starting point for further studies. Finally, despite that they did not undergo abdominal surgery operations, we chose to not consider neurosurgical patients together with cardiothoracic subjects as central nervous system has a specific regulation of vascular resistance that could be a confounding factor of our results; even they consisted only of two patients. For this reason we preferred to include them in the general population.

Our study has some limitations.

First, despite that our analysis found that RRI = 0.70 could be a good cut-off value to predict whether a patient will be exposed to the risk of any complication, we cannot say this is true for the entire population. A much larger sample is mandatory. Then we may consider our results as “preliminary results.”

Second, we enrolled a mixed population of patients undergoing different surgery; it may be a misleading factor.

Third, we considered cardiac-surgery population apart as patients had not surgically manipulated the splanchnic circulation, but we know the consequences of cardio-pulmonary circulation in terms of systemic inflammatory reaction, kidney injury, and haemodynamics.

In conclusion we may say that RRI > 0.70 is a helpful parameter to predict whether surgical population may experience any postoperative complications. The higher is the value the greater is the risk. Further investigations need to confirm or not our results.

## Figures and Tables

**Table 1:** Population characteristics.

Age (years)	61.7 ± 14.6
BMI (Kg/m^2^ body surface area)	25.4 ± 3.9
ASA 3-4 (*n*)	102 (49.76%)
Smoking (*n*)	98 (47.80%)
Diabetes (*n*)	26 (12.68%)
Hypertension (*n*)	100 (48.78%)
COPD (*n*)	43 (20.98%)
Myocardiopathy (*n*)	94 (45.85%)
Serum creatinine (mg/dL)	0.855 (0.44–2.0)
EF (%)*	54.6 ± 8.4
EDV (mL)*	154 (55–180)
Urine albumin (*n*)	16 (7.80%)
Systolic arterial pressure (SAP) mmHg^§^	130 ± 19
Diastolic arterial pressure (DAP) mmHg^§^	72 ± 12
Mean arterial pressure (MAP) mmHg^§^	88 ± 15
Heart Rate (bpm)^§^	75 ± 12
Haematocrit (%)	39.3 ± 5.4

*Only 96 patients underwent preoperative echocardiogram.

^§^Blood pressure and heart rate were recorded when patient arrived in operative room before any drug administration.

BMI: Body Mass Index; COPD: chronic obstructive pulmonary disease;

EF: ejection fraction; EDV: end-diastolic volume.

**Table 2:** Risk factors and RRI > 0.7.

	Global population			Cardiothoracic population		
	RRI < 0.7	RRI > 0.7	*P*	RRI < 0.7	RRI > 0.7	*P*
*N*	145	60		80	42	
Male (*n*)	101 (69.66%)	38 (63.33%)	0.378	57 (71.25%)	29 (69.05%)	0.800
Age (years)	60 ± 15	65 ± 13	0.045	60 ± 17	62 ± 13	0.388
BMI (Kg/m^2^ body surface area)	25.0 ± 3.9	26.4 ± 3.8	0.020	25.4 ± 4.1	26.7 ± 4.1	0.092
ASA (3-4)	65 (44.83%)	37 (61.67%)	0.028	48 (60.00%)	28 (66.67%)	0.470
Smoking (*n*)	62 (42.76%)	36 (60.00%)	0.025	32 (40.00%)	26 (61.90%)	0.021
Diabetes (*n*)	13 (8.97%)	13 (27.67%)	0.020	7 (8.75%)	6 (14.29%)	0.367
Hypertension (*n*)	65 (44.83%)	35 (58.33%)	0.078	35 (43.75%)	23 (57.76%)	0.247
COPD (*n*)	28 (19.31%)	15 (25.00%)	0.363	14 (17.50%)	7 (16.67%)	1.000
Myocardiopathy (*n*)	61 (42.07%)	33 (55.00%)	0.091	41 (51.25%)	23 (54.76%)	0.712
Serum creatinine (mg/dL)	0.90 ± 0.38	1.08 ± 0.60	0.005	0.96 ± 0.49	1.17 ± 0.69	0.029
EF (%)	56 ± 8	53 ± 8	0.127	56 ± 9	53 ± 9	0.149
EDV (mL)	151 ± 21	133 ± 35	0.049	156 ± 9	138 ± 34	0.124
Urine albumin (*n*)	8 (5.52%)	8 (13.33%)	0.083	5 (6.25%)	5 (11.90%)	0.310
Systolic arterial pressure (SAP) mmHg	130 ± 18	131 ± 21	0.813	129 ± 17	125 ± 17	0.274
Diastolic arterial pressure (DAP) mmHg	73 ± 11	71 ± 12	0.164	73 ± 10	69 ± 11	0.013
Mean arterial pressure (MAP) mmHg	89 ± 15	87 ± 17	0.204	87 ± 14	82 ± 13	0.058
Heart Rate (bpm)	76 ± 13	74 ± 12	0.518	76 ± 12	75 ± 11	0.744
Haematocrit (%)	39.9 ± 5.2	37.8 ± 5.7	0.030	40 ± 5	37 ± 6	0.014
Surgical APGAR score (points)	7 (1–10)	7 (1–10)	0.520	6 (1–10)	6.5 (1–10)	0.913

Blood pressure and heart rate were recorded when patients arrived in operative room but before any drug administration.

Surgical Apgar Score was calculated at the end of operation as it considers three items that have to be recorded after surgery completion.

**Table 3:** Outcome results and RRI.

	Global population			Cardiothoracic population		
	RRI < 0.7	RRI > 0.7	*P*	RRI < 0.7	RRI > 0.7	*P*
*N*	145	60		80	42	
Global complications	27 (18.62%)	19 (31.67%)	0.042	14 (17.50%)	15 (35.71%)	0.042
Suture leak	2 (1.38%)	2 (3.33%)	0.582	0	0	
Bleeding	4 (2.76%)	1 (1.67%)	1.000	3 (3.75%)	0	0.550
ARDS	1 (0.69%)	3 (5.00%)	0.076	1 (1.25%)	3 (7.14%)	0.117
Pneumonia	4 (2.76%)	7 (11.67%)	0.016	1 (1.25%)	6 (14.29%)	0.007
Atelectasis	3 (2.07%)	3 (5.00%)	0.361	1 (1.25%)	3 (7.14%)	0.117
SIRS/Sepsis	2 (1.38%)	4 (6.67%)	0.062	2 (2.50%)	4 (9.52%)	0.180
Septic shock	1 (0.69%)	6 (10.00%)	0.003	0	4 (9.52%)	0.013
ARF	2 (1.38%)	8 (13.33%)	0.001	2 (2.50%)	7 (16.67%)	0.008
Pulmonary embolism	0	1 (1.67%)	0.293	0	1 (2.38%)	0.344
Acute respiratory failure	4 (2.76%)	2 (3.33%)	1.000	1 (1.25%)	2 (4.76%)	0.272
Hepatic insufficiency	1 (0.69%)	0	1.000	0	0	
Arrhythmia	8 (5.52%)	5 (8.33%)	0.530	6 (7.50%)	4 (9.52%)	0.735
ICU admission (*n*)	56 (38.62%)	24 (40.00%)	0.854	33 (41.25%)	18 (42.86%)	0.864
ICU staying (days)	1 (1–36)	4 (1–38)	0.001	3 (1–36)	5.5 (2–38)	0.006
Mechanical ventilation (*n*)	53 (36.55%)	19 (31.67%)	0.505	33 (41.25%)	17 (40.48%)	0.934
Mechanical ventilation (hours)	12 (1–240)	32 (6–300)	0.004	12 (1–240)	32 (6–300)	0.001
Length-of-stay (days)	8 (1–37)	8 (4–40)	0.246	8 (1–22)	8 (4–24)	0.023
Death (*n*)	2 (1.38%)	3 (5.00%)	0.151	1 (1.25%)	2 (4.76%)	0.272

Complications that did not occur are not reported. Some patients experienced more than 1 adverse event.

**Table 4:** RRI classes and complications risk.

	Global population (*n* = 205)	Cardiothoracic subgroup (*n* = 122)
RRI < 0.6	1	1
RRI 0.60–0.65	OR 1.34 (95% CI 0.39–4.75); *P* = 0.636	OR 1.02 (95% CI 0.17–6.06); *P* = 0.984
RRI 0.66–0.70	OR 1.92 (95% CI 0.57–6.51); *P* = 0.295	OR 1.38 (95% CI 0.25–7.67); *P* = 0.717
RRI 0.71–0.75	OR 2.93 (95% CI 0.79–10.9); *P* = 0.109	OR 3.38 (95% CI 0.59–19.4); *P* = 0.171
RRI > 0.75	OR 3.10 (95% CI 0.85–11.3); *P* = 0.087	OR 2.75 (95% CI 0.47–16.0); *P* = 0.260

When RRI > 0.7 OR triplicates, although it did not result to be statistically significant.
